# Comparative Pharmacokinetics of a Dual Inhibitor of HIV-1, NBD-14189, in Rats and Dogs with a Proof-of-Concept Evaluation of Antiviral Potency in SCID-hu Mouse Model

**DOI:** 10.3390/v14102268

**Published:** 2022-10-16

**Authors:** Cheryl A. Stoddart, Francesca Curreli, Stephen Horrigan, Andrea Altieri, Alexander V. Kurkin, Asim K. Debnath

**Affiliations:** 1 Division of Experimental Medicine, Department of Medicine, Zuckerberg San Francisco General Hospital, University of California, San Francisco, CA 94110, USA; 2 Laboratory of Molecular Modeling & Drug Design, New York Blood Center, Lindsley F. Kimball Research Institute, 310 E 67th Street, New York, NY 10065, USA; 3 Noble Life Sciences Inc., Woodbine, MD 21784, USA; 4 EDASA Scientific srls, Via Stingi 37, 66050 San Salvo, Italy

**Keywords:** human immunodeficiency virus type 1 (HIV-1), HIV-1 gp120 antagonist, dual inhibitors, pharmacokinetics (PK), bioavailability, SCID-hu mouse model

## Abstract

We earlier reported substantial progress in designing gp120 antagonists. Notably, we discovered that NBD-14189 is not only the most active gp120 antagonist but also shows antiviral activity against HIV-1 Reverse Transcriptase (RT). We also confirmed its binding to HIV-1 RT by X-ray crystallography. The dual inhibition is highly significant because, intriguingly, this compound bridges the dNTP and NNRTI-binding sites and inhibits the polymerase activity of isolated RT in the enzymatic assay. This novel finding is expected to lead to new avenues in designing a novel class of HIV-1 dual inhibitors. Therefore, we needed to advance this inhibitor to preclinical assessment. To this end, we report the pharmacokinetics (PK) study of NBD-14189 in rats and dogs. Subsequently, we assessed the toxicity and therapeutic efficacy in vivo in the SCID-hu Thy/Liv mouse model. The PK data indicated a favorable half-life (t_1/2_) and excellent oral bioavailability (%F = 61%). NBD-14189 did not show any measurable toxicity in the mice, and treatment reduced HIV replication at 300 mg/kg per day in the absence of clear evidence of protection from HIV-mediated human thymocyte depletion. The data indicated the potential of this inhibitor as an anti-HIV-1 agent and needs to be assessed in a non-human primate (NHP) model.

## 1. Introduction

Advances in available therapeutics, particularly combination antiretroviral therapy, have significantly improved the treatment of HIV infections, facilitating the shift from a disease characterized by high morbidity and mortality to a manageable chronic illness. Despite this remarkable success, current treatments suffer from several limitations, including (1) reliance on daily adherence, (2) long-term use resulting in long-term toxicity, (3) limited treatment options due to the development of drug resistance, (4) high costs, and (5) the failure of current treatments to eradicate HIV. In addition, despite tremendous efforts and investment, no effective vaccine or microbicide has been developed, and significant hurdles must be overcome to achieve an effective cure. Thus, the continued development of small-molecule drugs with high potency against novel targets and minimal side effects remains imperative. The development of novel therapeutics will increase the number of available drugs, extend the scope of combination therapies, and present opportunities to formulate long-acting drugs. Our group has made significant progress toward meeting this critical need by developing a new class of HIV-1 entry inhibitors targeting the Phe43 cavity of HIV-1 gp120 [1,2,3,4,5,6,7,8,9], a distinct binding site from that was targeted by the US Food and Drug Administration (FDA)-approved attachment inhibitor, fostemsavir (Rukobia, Viiv Healthcare) [10].

Approval of Fostemsavir (BMS-663068) [11,12], a prodrug attachment inhibitor developed by ViiV Healthcare / GlaxoSmithKline (initially discovered by Bristol Meyers & Squibb) in July 2020, validated HIV-1 gp120 as a prime target for developing novel drugs for HIV-1 patients who have limited choice of drugs for treatment. However, due to the void in any effective prophylactic vaccines, it is critically important that the drug design and development studies on HIV-1 continue. In 2005, we pioneered the identification of a small molecule HIV-1 inhibitor, NBD-556 [9], that specifically targets the Phe43 cavity of HIV-1 envelope glycoprotein gp120. We have confirmed its binding to HIV-1 gp120 by x-ray structure [8]. Furthermore, with the structural insight gained and through a successive design-structure-design sequence, we made substantial progress in designing NBD-14189, a small molecule with broad-spectrum antiviral activity against a large panel of diverse clinical isolates and improved selectivity index (SI) [2].

Studies performed to evaluate the binding mode of NBD-14189 have shown a 5-fold increase of the IC_50_ against a mutant pseudovirus HIV-1_HXB-2_ carrying the A204D amino acid substitution, located in the V2 of the Env gp120 region. Notably, A204D mutation was observed in the in vitro selection experiment with BMS-626529 [13]. In addition, we observed a 7-fold increase of the IC_50_ against a mutant pseudovirus carrying the I424F amino acid substitution located in the CD4-binding site [2].

**Figure 1:**
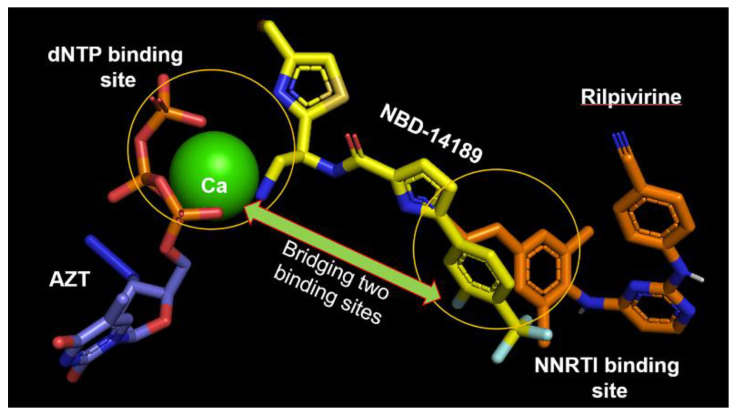
Superposition of RT/NBD-14189 (PDB: 7LPW [14]), RT/Riplivirine (PDB: 4G1Q [17]), and RT/AZT-TP/calcium ion (PDB: 5I42 [18]) shows that NBD-14189 bridges the binding site of dNTP and NNRTI.

Our recent discoveries reveal that some of the most active gp120 antagonists also display antiviral activity against HIV-1 reverse transcriptase (RT) [14]. We confirmed the binding of NBD-14189 with HIV-1 RT by X-ray crystallography (PDB:7LPW). This compound achieves dual inhibitory activity by bridging dNTP- and non-nucleoside RT inhibitor (NNRTI)-binding sites (Figure 1) to inhibit the polymerase activity of isolated RT in enzymatic assay [14]. Similar binding was reported for compound G from Merck [15] (PDB: 5VZ6) and dihydroxybenzoyl naphthyl hydrazone (DHBNH; PDB:2I5J) [16]. However, the structure indicated that NBD-14189 binding extends into the NNRTI-binding pocket on one end, toward the active polymerase site, and into the nucleotide-binding site on the other end, reaching further than compound G or DHBNH. Therefore, our studies suggested that this small molecule belongs to a novel class of dual-acting inhibitors [14].

Early in vitro ADMET (absorption, distribution, metabolism, excretion, and toxicity) and in vivo PK studies of drug molecules during drug discovery play a critical role in reducing the attrition rate during late-stage drug development [19,20,21,22,23,24]. In vitro ADMET studies are essential for initial drug profiling. However, in vivo PK studies involving various dosing routes are critical because they provide multifactorial results on the combined ADME effects [15,16,17,18,19,20,21,22,23,24,25,26,27,28]. In addition, in vivo studies will enable us to generate measurable PK data, which will be useful for medicinal chemistry optimization and clinical candidate nomination and development. Unfortunately, many drugs failed to reach the clinic with excellent efficacy due to poor ADMET pharmacokinetics (PK) profiles [29]. Therefore, two critical parameters in early drug design and development are desirable oral bioavailability (%F) and plasma half-life (t_1/2_). We performed a comparative ADME study of NBD-14189 with BMS-626529 (Temsavir) [30,31], revealing favorable drug-like characteristics to develop it further as a preclinical candidate. Consequently, we decided to evaluate the PK profile of NBD-14189 in Sprague Dawley rats and Beagle dogs as the next step. The dogs have been used as a preferred choice in assessing PK in many drug development studies due to their similarities in anatomical and metabolism profiles with humans.

The SCID-hu Thy/Liv mouse model, which we used to evaluate the antiviral potency and tolerability of NBD-14189, has been proven useful as a preclinical evaluation tool of antiviral efficacy in vivo [32,33,34,35,36,37,38]. The implant of human thymus in these mice supports the long-term differentiation of human T cells. We standardized and validated the model with four classes of US FDA-approved anti-HIV-1 drugs [32,33]. One significant advantage of this model is its high susceptibility to HIV infection (essentially 100%) after injecting a relatively small amount of infectious HIV-1 (1000 TCID_50_) directly into the thymus/liver implant.

This report aims to present the systematic use of PK profiles of NBD-14189 in rats and dogs and to evaluate its antiviral efficacy in the SCID-hu Thy/Liv mouse model. The data demonstrated the potential of this class of dual-acting inhibitors for further development in the treatment of HIV-1 infection.

## 2. Materials & Methods

### 2.1. PK Study in Rats and Dogs

Detail description is in the Supplementary Material.

### 2.2. Drugs and Viruses

NBD-14168 and NBD-14189 were reported earlier [2], and BMS-626529 was purchased from Apexbio, Boston, MA. Tenofovir disoproxil fumarate (TDF) and emtricitabine (FTC) were obtained from Gilead Sciences. HIV molecular clone pNL4-3 (CXCR4) from M. Martin [39] was obtained through the NIH AIDS Reagent Program, Division of AIDS, NIAID, NIH. To prepare the infectious supernatant HEK 293T cells were transfected using lipofectamine 2000. To calculate the stock virus titers, we infected human peripheral blood mononuclear cells (PBMCs) stimulated with phytohaemagglutinin (PHA) and assessed the supernatant p24 by endpoint dilution by enzyme-linked immunosorbent assay (ELISA) after 7 days. The 50% tissue culture infective doses (TCID_50_) were calculated using the Reed-Muench method.

### 2.3. In Vitro Antiviral Assay

The inhibitory activity of NBD-14189, NBD-14168 and BMS-626529 was evaluated in PHA-stimulated PBMCs pooled from 6 donors. Cryopreserved cells were thawed and cultured in RPMI 1640 supplemented with 10% heat-inactivated fetal bovine serum (FBS) and 5 U/mL of human recombinant interleukin-2 (rIL-2). The following day, the cells were inoculated in bulk with HIV at a multiplicity of infection (MOI) of 0.001 for 2 h at 37 °C. HIV-infected PBMCs were seeded in triplicate into round-bottom 96-well plates at 100,000 in 100 µL volume and treated with 100 µL of serially diluted NBD-14189, NBD-14168, BMS-626529, or medium alone (negative control) and cultured for 7 days. Supernatants were collected diluted 1:800 and assayed for p24 by quantitative HIV-1 p24 ELISA (PerkinElmer). The cytotoxicity assay was performed in parallel: uninfected PBMCs were treated with serially diluted compounds and incubated with (4,5-dimethylthiazol-2-yl)-2,5-diphenyl tetrasodium bromide (MTT) on day 7. Fifty percent inhibitory concentrations (IC_50_) and 50% cytotoxic concentrations (CC_50_) were calculated by a 4-parameter fit model (SOFTmaxPRO 3.0, Molecular Devices). At day 7, untreated virus control wells had mean HIV-1 p24 concentrations of 180–230 ng/mL.

### 2.4. SCID-hu Thy/Liv Mice

The animal facility located in the University of California, San Francisco (UCSF) is a facility accredited by the Association for Assessment and Accreditation of Laboratory Animal Care International (AAALAC). All animal experiments were conducted following the Guide for the Care and Use of Laboratory Animals and approved by the UCSF Institutional Animal Care and Use Committee. To generate the SCID-hu Thy/Liv mice 7–8-week-old male C.B17 SCID mice (model CB17SC-M, C.B-*Igh-1^b^*/IcrTac-*Prkdc^scid^*; Taconic, New York, NY, USA) were implanted under the kidney capsule with 1-mm^3^ pieces of human fetal thymus and liver from a single donor as described previously [32,33]. Twenty weeks after implantation, implants of anesthetized mice were inoculated by direct injection with 50 µL of HIV-1_NL4-3_ (1000 TCID_50_) or RPMI 1640 medium (mock infection). NBD-14189 was dissolved in 0.5% medium-viscosity carboxymethylcellulose (CMC, Sigma St. Louis, MO, USA) and administered to 6 mice per group at a range of dosage levels (30, 100, and 300 mg/kg of body weight/day). As a positive antiviral control, TDF plus FTC (Truvada; Gilead Sciences, Inc., Foster City, CA 94404, USA) were dissolved in 0.5% CMC and administered to 6 mice at a total dosage of 100 mg/kg/day (60 mg/kg/day TDF plus 40 mg/kg/day FTC). All mice were treated twice daily with PO of 200 µL beginning 7 days before HIV inoculation and continuing until Thy/Liv implant collection.

To collect the Thy/Liv implants, mice were euthanized 21 days after inoculation, at the time HIV_NL4-3_ replication was expected to peak in the implants. Mice were euthanized by cervical dislocation following terminal blood collection. Single-cell suspensions were made by placing each Thy/Liv implant into a sterile nylon mesh bag, submerging the bag in PBS containing 2% FBS, and dispersing the tissue between the nylon layers with forceps as described previously [33,34,35]. For quantification of viral RNA, total RNA was extracted from thawed implant cells using the AllPrep DNA/RNA mini kit (Qiagen Hilden, Germany), and RNA was eluted in 25 μL nuclease-free water (Ambion Austin, TX, USA) into DNA LoBind tubes (Eppendorf, Hamburg, Germany). The HIV RNA standard curve was generated using 10-fold serial dilutions of premeasured HIV RNA extract from NL4-3 culture supernatant, and HIV RNA copies were normalized to the RPLP0 (large ribosomal protein) standard curve extracted from fresh unstimulated PBMCs. Two separate qPCR mixes were prepared for HIV copy number and RPLP0 copy number. Quantitative PCR (q-PCR) samples were prepared in a 20-μL reaction volume containing 5 μL eluted sample, 0.5 μL TaqMan RT enzyme mix (40×), 10 μL TaqMan RNA-to-CT 1-step kit (2×), 0.4 μL PrimeTime primer/probe set or 1 μL RPLP0 primer/probe set (20×), and nuclease-free water. Thermocycling conditions were 15 min at 48 °C and 10 min at 95 °C, followed by 60 cycles of 95 °C for 15 s and 60 °C for 30 s for HIV PCR; or 40 cycles of 95 °C for 15 s and 60 °C for 30 s for RPLP0 PCR. The HIV copy number proportional to the cycle threshold (CT) was calculated using the standard curve and converted into log_10_ copies per well, then divided by the number of RPLP0 copies per well and multiplied by 1,000,000 to estimate HIV RNA copies per 10^6^ human cells and finally, the log_10_ values were used for calculation of geometric means. The limit of detection was 2 HIV RNA copies per 10^6^ human cells, and this lower-limit value for samples with undetectable viral RNA was used for the calculation of the mean. For p24 quantitative ELISA, pellets of 2.5 × 10^6^ cells were lysed with 400 µL of p24 lysing buffer (1% Triton X-100, 0.5% sodium deoxycholate, 5 mM EDTA, 25 mM Tris Cl, 250 mM NaCl, and 1% aprotinin) and rotated overnight at 4 °C, then preserved at −20 °C. ELISA was performed by transferring thawed samples into HIV-1 p24 antibody-coated microplates obtained from PerkinElmer and following the manufacturer’s instructions. A standard curve was generated with the kit-supplied standards, and the results were calculated as pg p24 per 10^6^ implant cells. The limit of detection of the p24 ELISA was 5 pg p24 per 10^6^ cells.

### 2.5. Flow Cytometry

Thy/Liv implant cells were fixed and permeabilized with 1.2% paraformaldehyde and 0.5% Tween 20. Pellets containing 10^6^ cells were resuspended in 50 μL of PBS containing 0.8 mg/mL human IgG (Biodesign International) and a mixture of monoclonal antibodies: phycoerythrin cyanine dye CY7-conjugated anti-CD4 (BD Biosciences), cyanine dye CY5.5-conjugated anti-CD8 (Invitrogen), and allophycocyanin E fluor 780 dye-conjugated anti-CD3 (eBiosciences). As a control for nonspecific antibody binding, cells from one implant were also stained with conjugated isotype-matched antibodies. Cells were incubated for 30 min in the dark and washed two times with PBS/2% FBS, resuspended in 200 μL of PBS/2% FBS, and analyzed on an LSR II instrument (BD Biosciences). Optimization of fluorescence compensation for correction of fluorescence spectral overlaps emitted from the fluorescently conjugated antibodies was achieved by staining cells with each antibody alone plus anti-mouse Ig kappa chain and negative control BD CompBeads (BD Biosciences), as directed by the manufacturer. A total of 100,000 live cell events were collected for each sample. The percentages of marker-positive (CD4+, CD8+, and double-positive, DP) thymocytes in the implant samples were determined by first gating on a live lymphoid cell population identified by forward- and side-scatter characteristics and then by CD3 expression, as described previously [35]. CD4/CD8 ratios were calculated by dividing the percentage of CD4+ cells by the percentage of CD8+ cells for each individual implant.

## 3. Results and Discussions

### 3.1. Pharmacokinetic Study in Rat

To determine bioavailability and exposure of NBD-14189, Sprague Dawley rats were dosed by intravenous (IV) injection and by oral gavage (PO). Blood samples were collected over a 24 h period, and plasma drug levels were determined. Animals were dosed by PO or by IV injection through the tail vein with NBD-14189 at 10 mg/kg, and blood was collected at 0.25, 0.5, 1, 2, 4, 8, and 24 h post PO dose, and 0.08, 0.25, 0.5, 1, 2, 4, 8, and 24 h for IV dose, and blood plasma was isolated.

**Figure 2:**
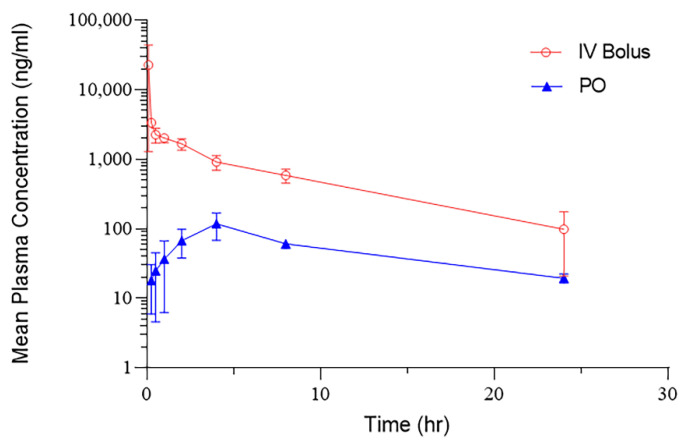
Pharmacokinetics of NBD-14189 in the Rat model. NBD-14189 was dosed by IV bolus or PO administration at 10 mg/kg, and the concentration of the compound in plasma was determined at the indicated time points.

Pharmacokinetic analysis of NBD-14189 showed peak drug levels (C_max_) at 0.08 h for IV administration and 8 h for PO administration (Figure 2). A Half-life (T_1/2_) of 9.8 h was calculated for the IV dose. The half-life for the PO dose was 8.19 h, similar to the intravenous dose clearance (Table 1). The oral bioavailability of NBD-14189 in a methylcellulose suspension showed limited drug plasma levels at this dose, with the mean percent bioavailability (%F) of 6.7%.

### 3.2. Pharmacokinetic Study in Dogs

Due to inconclusive data from the rat PK study, especially regarding t_1/2_ and poor bioavailability, we decided to move the PK study to a dog model. This study aimed to evaluate the PK and oral bioavailability of NBD-14189 when administered as a single IV or PO dose to non-naïve male Beagle dogs. The primary reason for selecting single-sex to determine single-dose PK in early-stage programs is consistent with the imperative to reduce animal use. Males have been the industry standard for single-sex experimentation based on traditional views of hormonal stability and body composition (muscle-to-fat ratio, etc.). Depending on the therapeutic target of the drug, PK and toxicokinetic in both sexes will have to be determined as the program advances. Sex differences sometimes manifest with repeated dosing as metabolizing enzymes are differentially stimulated, so efficacy and toxicity profiles can vary over time.

This multiphase study consisted of two treatment groups (Groups 1 and 2) of three dogs. Dogs were administered 1 mg/kg NBD-14189 (Group 1) once via IV at a dose volume of 1 mL/kg in Phase 1, and 2 mg/kg NBD-14189 (Group 2) once via PO administration at a dose volume of 2 mL/kg in Phase 2.

Physical examinations were recorded at the time of randomization. We recorded clinical observations on Days 1–3 of each phase. Body weight measurements were taken for randomization and before dose administration during each phase. Plasma samples were collected from both groups at 0.083 (Phase 1 only), 0.25, 0.50, 1, 2, 4, 8, 24, 30, and 48 h post dose per phase for analysis of systemic exposure to NBD-14189. This study had no abnormal clinical observations and no test article-related effects on body weight.

PK summary data are shown in Table 2. The summary of bioavailability data for NBD-14189 is shown in Table 3.

Following a single 1 mg/kg IV bolus or 2 mg/kg PO administration of NBD-14189 in male Beagle dogs, NBD-14189 was quantifiable in all animals throughout the entire 48 h sampling interval. The mean initial concentration (C_0_) was 1230 ng/mL, mean peak (C_max_) and total (AUC_last_) exposures were 844 ng/mL and 2600 h*ng/mL, respectively, for animals administered IV bolus and 181 ng/mL and 3020 h*ng/mL, respectively, for animals, administered PO. Mean half-life (T_1/2_) and AUC_INF_ values were 20.0 h and 3130 h*ng/mL, respectively, for IV bolus administration, and 24.3 h and 3820 h*ng/mL, for PO administration. Mean clearance (Cl) was 5.77 mL/min/kg, and the mean volume of distribution based on the terminal phase (Vz) was 9.60 L/kg in animals administered IV bolus. Following PO administration of NBD-14189, the mean percent bioavailability (%F) was calculated to be 61.0%, which was about 10-fold higher than observed in rats. Although we do not know the reason for this vast anomaly, different formulations and doses may have played some role. 

**Figure 3:**
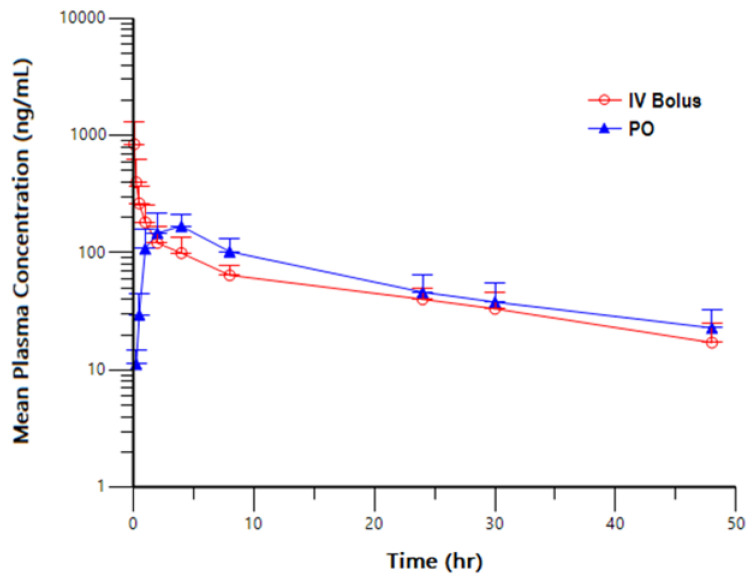
Mean NBD-14189 Plasma Concentration versus Time profiles following a single IV bolus or PO administration of NBD-14189 in male Beagle Dogs.

A plot of mean NBD-14189 Plasma Concentration versus Time profiles following a single IV bolus or PO administration of NBD-14189 in male Beagle Dogs has been shown in Figure 3.

### 3.3. Evaluation of the Anti-HIV-1 Activity of NBD-14189 against NL4-3 Virus in SCID-hu Thy/Liv Mice

Due to the measurable progress, we made in developing NBD-series HIV-1 gp120 antagonists, and excellent antiviral potency of NBD-14189, and the high oral bioavailability in dogs led us to pursue a proof-of-concept antiviral efficacy study in SCID-hu Thy/Liv mouse model. However, before starting this animal study, we wanted to ensure that the inhibitor shows efficacy in the same virus (HIV-1_NL4-3_) in PBMCs that would be used for the in vivo efficacy study.

#### Evaluation of the Antiviral Activity against HIV-1_NL4-3_ in PHA-Stimulated Human PBMCs

**Figure 4:**
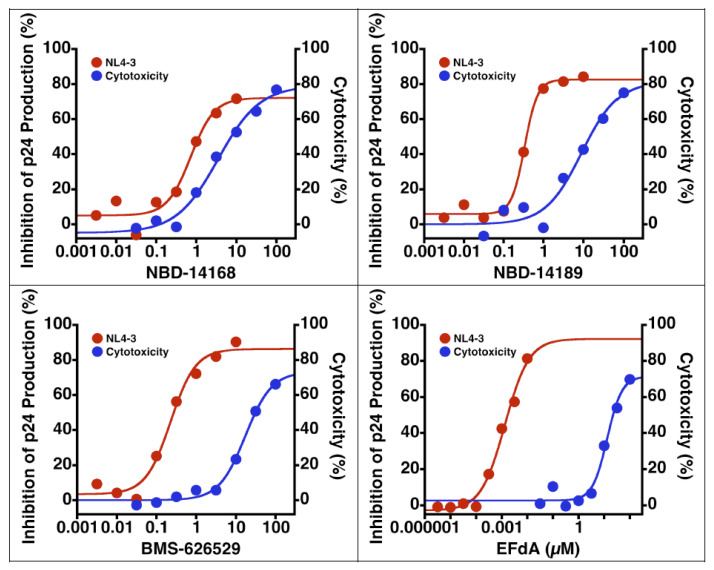
Dose–response plots of in vitro antiviral efficacy and cytotoxicity in PHA-stimulated human PBMCs.

Before starting in vivo antiviral efficacy study in the SCID hu-mouse model, we wanted to test NBD-14189 in vitro against HIV-1_NL4-3_ in PHA-stimulated PBMCs. Therefore, two phenyl ring-containing active analogs, NBD-14168 [2] and NBD-14189 [2], were selected for the assay. In addition, BMS-626529 [40], an attachment inhibitor, and EFdA [41], a reverse transcriptase translocator inhibitor, were chosen as positive controls. The data in Table 4 show that both the NBD compounds have excellent antiviral potency, especially NBD-14189, which had similar activity to BMS-626529 that also targets HIV-1 gp120. However, the cytotoxicity of the BMS compound was about 3-fold better. The dose–response plots are shown in Figure 4.

### 3.4. NBD-14189 Tolerability and Plasma Levels in SCID-hu Thy/Liv Mice

**Figure 5:**
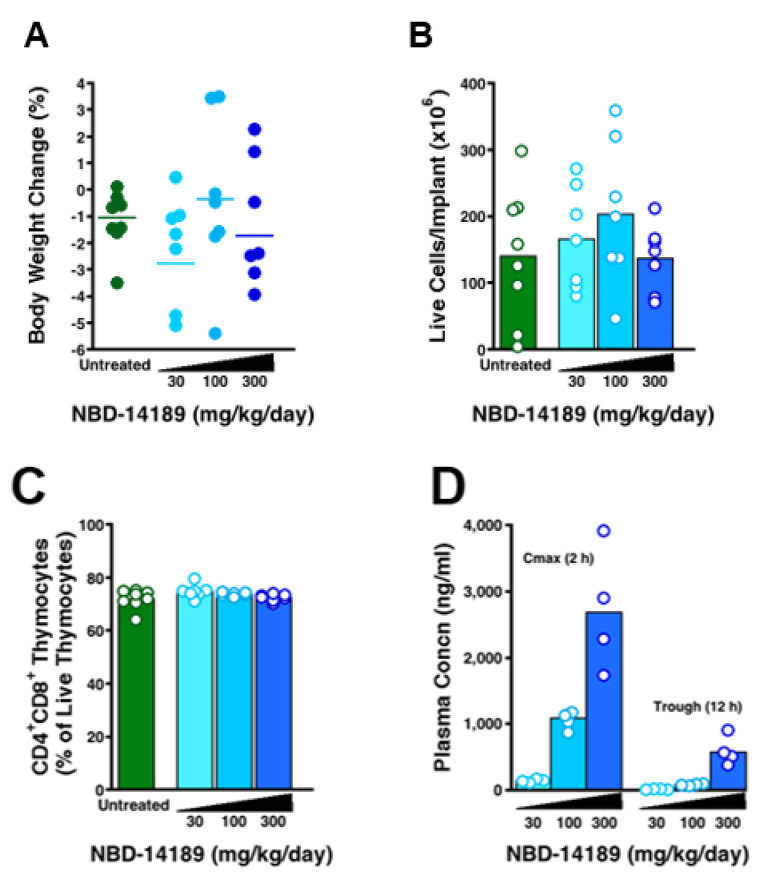
Treatment of SCID-hu Thy/Liv mice with NBD-14189 was well tolerated with no change in body weight (**A**), no apparent deleterious effects on Thy/Liv implant cellularity (**B**) or CD4^+^CD8^+^ thymocytes (**C**), and high C_max_ NBD-14189 plasma concentrations (**D**). The columns represent the means, and the open circles represent individual mice from the same mouse cohort. Values for treated groups were not statistically significant (*p* > 0.05) from untreated mice by the Mann-Whitney U test.

Groups of 6 mice each were treated with NBD-14189 by twice-daily PO for 21 days at dosage levels of 30, 100, and 300 mg/kg/day to determine an appropriate dosage range for a subsequent anti-HIV efficacy study. All three dosage levels were well tolerated with no significant change in body weight compared to untreated mice (Figure 5A) and no significant changes in the total cellularity of their Thy/Liv implants (averaging 140–200 × 10^6^ cells per implant across groups) or percentage of immature cortical CD4^+^CD8^+^ thymocytes (averaging 72–75% of live thymocytes). Terminal blood collected from treated mice 2 h after the last dose had mean plasma C_max_ levels of 147, 1055, and 2713 ng NBD-14189 per mL at dosages of 30, 100, and 300 mg/kg/day, respectively (Table 5). Plasma NBD-14189 trough levels 12 h after the last dose was 17, 92, and 590 ng/mL, respectively. These results show that NBD-14189 has good bioavailability after oral dosing with a prolonged in vivo half-life seemingly sufficient for potent in vivo antiviral activity.

In this first-in-mouse study, NBD-14189 was well tolerated in SCID-hu Thy/Liv mice at doses up to 300 mg/kg/day over 21 days of twice-daily oral dosing (Figure 5). Treatment had no significant effect on body weight (Figure 5A), and there were no apparent effects on the generation of human T-cells in their Thy/Liv implants (Figure 5B,C). Moreover, mice had both high C_max_ and trough levels of NBD-14189 in their plasma 2 and 12 h after dosing, respectively (Figure 5D). The mean C_max_ at the highest dose was 2713 ng/mL (Table 5), and this plasma level would be expected to exert an antiviral effect given that the in vitro IC_50_ in human PBMCs is 0.38 µM (163 ng/mL). 

### 3.5. NBD-14189 in HIV-Infected SCID-hu Thy/Liv Mice 

**Figure 6:**
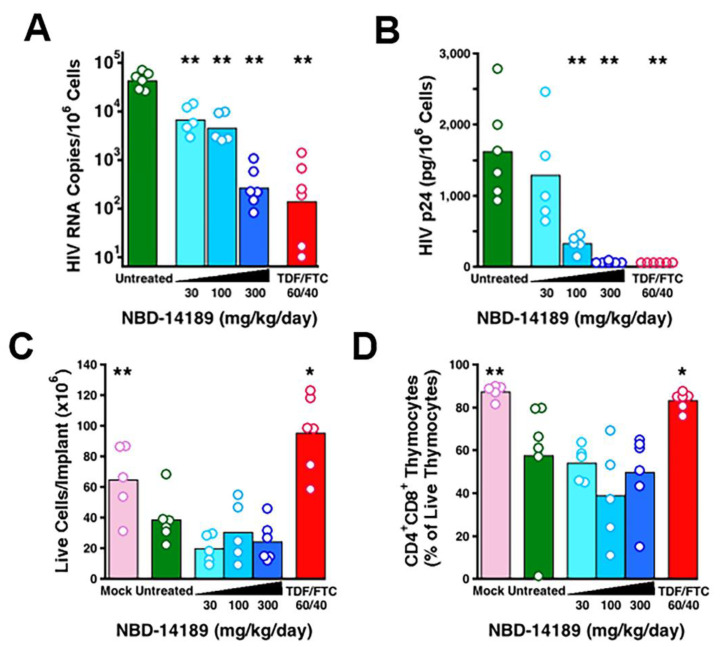
Treatment of SCID-hu Thy/Liv mice with NBD-14189 caused dose-dependent reductions in HIV RNA (**A**) and undetectable p24 (**B**) in 5 of 6 mice at 300 mg/kg/day. Despite this apparent evidence of antiviral activity by treatment with NBD-14189, there was no apparent protection from HIV-mediated reductions in total implant cellularity (**C**) or from virus-mediated depletion of CD4^+^CD8^+^ thymocytes (**D**) 21 days after inoculation. *, *p* < 0.05; **, *p* < 0.01 (compared to untreated mice by Mann–Whitney U test).

In a proof-of-concept first evaluation, we administered NBD-14189 to SCID-hu Thy/Liv mice by twice-daily PO beginning 7 days before HIV_NL4-3_ inoculation and continuing until implant collection 21 days after inoculation (for a total dosing period of 28 days). All 6 untreated mice had high levels of HIV RNA (mean of 4.6 log_10_ copies per 10^6^ cells; Figure 6A) and HIV p24 capsid protein (mean of 1600 pg per 10^6^ cells; Figure 6B) in their implants. As expected for this cytopathic CXCR4 HIV strain, HIV_NL4-3_ infection caused statistically significant reductions in implant cellularity (mean of 39 × 10^6^ versus 65 × 10^6^ cells; Figure 6C), and CD4^+^CD8^+^ thymocytes (mean 58% versus 87%; Figure 6D) compared to mock-infected implants. Treatment with NBD-14189 caused dose-dependent reductions in both HIV RNA and p24, with 5 of 6 mice having undetectable p24 at the highest dosage level (300 mg/kg/day) and statistically significant reductions in HIV RNA at all three dosage levels compared to untreated mice.

Despite this apparent potent antiviral activity, the human thymocytes in these mice were not protected from HIV-mediated depletion as would be expected and as we have shown previously [33] and for the TDF/FTC-treated mice in this study. This does not seem to be the result of the toxicity of NBD-14189 to the human thymocytes because we saw no evidence of deleterious effects of treatment on Thy/Liv implant cellularity or thymocyte subsets in the tolerability study (Figure 5).

By comparison, treatment with TDF plus FTC resulted in undetectable p24 in all 6 mice, a mean reduction in viral RNA of 2.4 log10, and complete protection from virus-mediated thymocyte depletion.

Despite these mixed results, we have demonstrated that NBD-14189 has potent anti-HIV activity in human PBMCs in vitro, and it is well-tolerated in humanized mice after repeated daily dosing. The lack of protection from HIV-mediated thymocyte depletion was unexpected, given the large reductions in viral load after NBD-14189 treatment. Despite the apparent lack of protection in this one mouse study, NBD-14189 has promising characteristics that should be evaluated in further studies, perhaps in SIV-infected macaques, to fully explore its potential for HIV prevention and therapy.

## 4. Conclusions

As part of the preclinical evaluation of one of the most active gp120 antagonists and dual inhibitors (targeting both HIV-1 gp120 and RT), NBD-14189, we initiated PK studies in both rats and dogs. The PK data indicated drug-like characteristics of NBD-14189. However, the rat’s bioavailability data was inconclusive, leading us to evaluate PK in dogs. The bioavailability data in dogs was excellent (%F = 61). These encouraging data motivated us to test this inhibitor against HIV in the SCID-hu Thy/Liv mouse HIV model. NBD-14189 was well tolerated even at the highest dose (300 mg/kg per day body weight). At this highest dose, NBD-14189 treatment resulted in reductions of HIV RNA and p24 protein similar to that of TDF/FTC treatment at 60/40 mg/kg/day. Surprisingly, despite such seemingly potent antiviral activity, NBD-14189 did not protect against HIV-mediated depletion of human thymocytes in these mice, indicating a more thorough investigation is needed. However, we could not perform a more in-depth follow-up study because of the defunding of the Federal Contract that supported the use of the SCID-hu Thy/Liv mouse model for HIV therapeutics development. Despite this limitation, the proof-of-concept antiviral and tolerability data of NBD-14189 was promising enough to be moved to efficacy study in an SIV-infected macaque model soon.

## Figures and Tables

**Table 1:** Summary of pharmacokinetics parameters following a single IV bolus or PO administration of NBD-14189 in Rats.

PK Parameters	IV	PO	Unit
C_0_	58,750		ng/mL
C_max_	22,690	118	ng/mL
T_1/2_	7.58	8.19	H
AUC	22,076	1482	ng/mL*h
CL	0.0005	0.0067	(mg/kg)/(ng/mL)/h
Vss	0.003		(mg/kg)/(ng/mL)

Abbreviations: C_0_ = concentration immediately after initial; C_max_ Observed maximum plasma or serum concentration after administration (first) injection; T_1/2_ = time it takes for half the drug concentration to be eliminated; AUC = area under the plasma concentration-time curve; Cl = a proportionality factor that relates the concentration of drug measured in the body to the rate of elimination; V_ss_ = volume distribution at steady state. *, represent multiplication.

**Table 2:** Summary of NBD-14189 pharmacokinetics parameters following a single IV bolus or PO administration of NBD-14189 in male Beagle Dogs.

Dose Level (mg/kg)	Analyte	Route	C_0_ (ng/mL)	T_max_ (h)	C_max_ (ng/mL)	T_last_ (h)	AUC_last_ (h*ng/mL)	T _1/2_ (h)	AUC_INF_ (h*ng/mL)	Cl (mL/min/kg)	Vz (L/kg)
1	NBD-14189	IV Bolus	1230	0.083	844	48	2600	20.0	3130	5.77	9.60
2	NBD-14189	PO	-	3.3	181	48	3020	24.3	3820	-	-

Note: - = no data. Abbreviations: C_0_ = concentration immediately after initial; T_max_ = time to reach C_max_; C_max_ Observed maximum plasma or serum concentration after administration (first) injection; T_last_ = Time of Last Measurable Concentration; T_1/2_ = time it takes for half the drug concentration to be eliminated; AUC_last_ = area under the plasma concentration-time curve to the last measurable plasma concentration; AUC_inf_ = a theoretical measure of the total exposure of the drug to the body from administration till all the drug is eliminated; Cl = a proportionality factor that relates the concentration of drug measured in the body to the rate of elimination; V_z_ = volume distribution. *, represent multiplication.

**Table 3:** Bioavailability of NBD-14189 following a single IV bolus or PO administration in male Beagle Dogs.

Analyte	Dose Level (mg/kg)	Route	AUC_inf_/Dose (h*kg*ng/mL/mg)	F(%)
NBD-14189	1	IV Bolus	3130	-
NBD-14189	2	PO	1910	61.0

Note: - = no data. AUC_inf_ = a theoretical measure of the total exposure of the drug to the body from administration till all the drug is eliminated; (%F) = mean percent bioavailability. *, represent multiplication.

**Table 4:** Antiviral activity against HIV-1_NL4-3_ in PHA-stimulated human PBMCs.

Drug	NL4-3 Antiviral (PBMC) (µM)		PBMC Toxicity (µM)		SI
	IC_50_	IC_90_	CC_50_	CC_90_	CC_50_/IC_50_
NBD-14168	1.2	>10	7.6	>100	6.4
NBD-14189	0.38	>10	15	>100	39
BMS-626529	0.28	>10	32	>100	110
EFdA	0.0017	>0.066	23	>100	13,000

**Table 5:** Pharmacokinetics parameters of NBD-14189 in SCID-hu Thy/Liv mice.

Dose	C_max_	AUC_0-t_
300 mg/kg	2713	22,916
100 mg/kg	1055	8509
30 mg/kg	147	1196

## Data Availability

Data can be available from the corresponding authors upon request.

## Supplementary Materials

The following supporting information can be downloaded at: https://www.mdpi.com/article/10.3390/v14102268/s1, 1. PK study in rats; 2. PK study in Beagle Dogs; Table S1: Mann-Whitney U Test on treatment comparison data of NBD-14189 at different doses for toxicity evaluation in SCID-hu Thy/Liv mice; Table S2: Mann-Whitney U Test on treatment comparison data of NBD-14189 at different doses for antiviral efficacy evaluation in SCID-hu Thy/Liv mice.
