# Supplementary material for: Comparative Pharmacokinetics of a Dual Inhibitor of HIV-1, NBD-14189, in Rats and Dogs with a Proof-of-Concept Evaluation of Antiviral Potency in SCID-hu Mouse Model
Source: Viruses. 2022 Oct 16;14(10):2268. doi: 10.3390/v14102268 (PMC9611370; doi:10.3390/v14102268)
Supplement: Supplementary file 1 [file viruses-14-02268-s001.zip › viruses-1952248-supplementary.pdf]

Comparative Pharmacokinetics of a Dual Inhibitor of HIV-1, NBD-14189, in Rats and Dogs with a Proof-of-Concept Evaluation of Antiviral Potency in SCID-hu Mouse Model

Cheryl A. Stoddart [1,*], Francesca Curreli [2], Stephen Horrigan [3], Andrea Altieri [4], Alexander V Kurkin [4] and Asim K Debnath [2,*]

1 Division of Experimental Medicine, Department of Medicine, Zuckerberg San Francisco General Hospital, University of California, San Francisco, CA 94110, USA
2 Laboratory of Molecular Modeling & Drug Design, New York Blood Center, Lindsley F. Kimball Research Institute, 310 E 67th Street, New York, NY 10065, USA
3 Noble Life Sciences Inc., Woodbine, MD 21784, USA
4 EDASA Scientific srls, Via Stingi 37, 66050 San Salvo, Italy
* Correspondence: cheryl.stoddart@ucsf.edu (C.A.S.); adebnath@nybc.org (A.K.D.)

Supplemental Materials

PK study in rats

Six (6) Sprague Dawley female rats (three animals per group) were implanted with a jugular vein catheter for blood collection. For the preparation of oral dose solution, NBD-14189 was added to a final concentration of 8 mg/ml to a solution of 0.5% carboxymethylcellulose in sterile water and sonicated for 10 minutes at room temperature. For the preparation of the intravenous doing solution, NBD-14189 was dissolved in NMP at 80 mg/ml. A solution of 44% PEG400 in sterile water was added to give a final concentration of 8 mg/ml NBD-14189, 10% NMP and 40% PEG400. The solution was used within one hour of preparation. The compound was dosed within one hour of preparation. Animals were dosed by oral gavage (PO) using a feeding needle or by intravenous (IV) injection through the lateral tail. Blood was collected from the indwelling catheter into K2EDTA coated tubes at 0.25, 0.5, 1, 2, 4, 8, and 24 hours post PO dose, and 0.08, 0.25, 0.5, 1, 2, 4, 8, and 24 hours for IV dose. Samples were mixed well, and plasma was separated by centrifugation at 3000xg for 6 minutes. Plasma was collected and frozen at -80ºC until analyzed.

The concentrations of NBD-14189 in plasma were determined using high-performance liquid chromatography with tandem mass spectrometric detection (LC-MS/MS). NBD-14189 was isolated from plasma by liquid-liquid extraction. A partial aliquot of the supernatant was transferred to a clean 96-well collection plate, evaporated to dryness under nitrogen, and reconstituted with water. The extracted samples were analyzed using a Sciex 5500 mass spectrometer. The quantitative range of the assay was from 1-2,000 ng/mL. Pharmacokinetic parameters were analyzed using PkSolver[43]. Graphs were generated using Prism 9.2 from GraphPad Software, Sandiego, CA.

PK study in Beagle Dogs

This non-clinical laboratory study was exploratory in nature. It was not conducted in accordance with the principles outlined in the United States Food and Drug Administration (FDA) Good Laboratory Practice (GLP) Regulations, 21 Code of Federal Regulations (CFR) Part 58.

This study was conducted according to the Protocol (included with this report) and applicable Standard Operating Procedures of Inotiv. The in-life portion of this study was conducted by Inotiv staff at the Department of Comparative Medicine, St. Louis University.

The purpose of this study was to evaluate the pharmacokinetics (PK) and oral bioavailability of NBD-14189 when administered as a single intravenous (IV) or oral (PO) doses to non-naïve male beagle dogs.

This multiphase study consisted of two treatment groups (Groups 1 and 2) comprised of three male Beagle dogs each. Dogs were administered 1 mg/kg NBD-14189 (Groups 1) once via IV at a dose volume of 1 mL/kg in Phase 1, and 2 mg/kg NBD-14189 (Groups 2) once via PO gavage at a dose volume of 2 mL/kg in Phase 2.

Physical examinations were recorded at the time of randomization. Clinical observations were recorded on Days 1–3 of each phase. Body weight measurements were taken for randomization and prior to dose administration during each phase. Plasma samples were collected from both groups at 0.083 (Phase 1 only), 0.25, 0.50, 1, 2, 4, 8, 24, 30, and 48 hours post dose per phase for analysis of systemic exposure to

There were no abnormal clinical observations and no test article-related effects on body weight during this study.

All study-related activities were performed in accordance with the IACUC-approved Animal User Protocol SLU-2544.

Test Article Preparation

Phase 1 IV Formulation: NBD-14189 in 10%NMP/44% PEG400/SWFI v/v/v (solution)

Phase 2 PO Formulation: NBD-14189 in 0.5% Carboxymethylcellulose (Suspension)

Vehicle Preparation

Phase 1 IV Vehicle: 44% PEG400/SWFI v/v (solution)

Phase 2 PO Vehicle: 0.5% Carboxymethylcellulose in DI water (w/v) (solution)

Route, Frequency, and Duration of Administration

The administration was once per phase by PO gavage or IV injection.

Oral (PO) Dosing: Oral gavage dosing of liquid aqueous drug formulation was performed without anesthesia using manual restraint. One technician restrained the dog, and a second technician inserted the gavage tube and administered the dosing solution. The gavage tube was inserted through the mouth into the stomach. Care was taken so that the tube did not enter the trachea or puncture the esophagus or stomach. The length of the gavage tube was cut to match the distance from the stomach to the tip of the nose, and the outside of the tube was dipped in water to facilitate insertion. As the tube approached the pharynx, the neck was gently extended to facilitate introduction into the stomach. Once properly placed, the content was dispensed, and the gavage tube was removed. Compounds administered via PO gavage injections were administered at a volume of 2 mL/kg. Administration of test compounds occurred on Day 1, and the duration of dose administration was a single dose per phase.

IV Injections: IV injections were performed without anesthesia using manual restraint. One technician restrained the dog, and a second technician performed the IV injection. IV dosing

formulation components were prepared aseptically and injected via direct venipuncture of the cephalic (primary). The veins were manually occluded, the needle (20–25 g) was inserted parallel to the vein, and the tip was directed into the lumen along the longitudinal axis. Compounds administered via IV injections were administered at 1 mL/kg (single dose). When dosing was completed, the injection site was occluded with a gauze pad by the technician for an appropriate period (30 seconds to one minute) to ensure hemostasis was achieved.

Following IV dose administration, the cephalic vein was used for blood sample collection 0.083 (IV only), 0.25, 0.50, 1, 2, 4, 8, 24, 30, and 48 hours post dose. Samples were collected in K_2EDTA tubes and centrifuged under refrigerated conditions to collect plasma. The plasma was frozen at -70°C until transferred for bioanalysis. Samples were analyzed by LC-MS/MS using 0.1% Formic Acid in Water (Mobile Phase A) and 0.1% Formic Acid in Acetonitrile (Mobile Phase B) in a Restek Raptor Biphenyl (2.1 x 30 mm, 2.7 µm) column.

Pharmacokinetic Evaluation

Pharmacokinetic analyses were performed on the individual plasma concentration versus time data for NBD-14189 using Phoenix WinNonlin software (Version 8.1 or higher) non-compartmental analysis (linear trapezoidal rule for AUC calculations). Nominal dose values and nominal sampling times were used for calculations. Any concentrations reported as BLQ were set equal to zero. For calculations of AUC on Study Day 1, the plasma levels of NBD-14189 at time zero were set equal to zero. The pharmacokinetic analysis included the determination of %F, C_{max}, T_{max}, AUC_{last}, AUC_{INF}, $T_{1/2}$ for IV/Oral; C_0, Vz and Cl for IV. Mean NBD-14189 plasma concentrations versus time data were presented and reported to three significant figures and CV% to one decimal place. Pharmacokinetic parameters were presented as individual and mean values. Individual T_{max} and T_{last} were reported to two significant figures, while all other values were reported to three significant figures. Mean NBD-14189 PK parameters, except for $T_{max,}$ and T_{last} were

presented to three significant figures and CV% to one decimal place. Mean T_{max} and T_{last} values were presented to two significant figures.

Absolute bioavailability was calculated for the mean PO route of administration using the mean IV administration as a test reference correcting for dose. Calculations of bioavailability were performed using the following equation: $\%F = AUC_{INF,\,PO} * Dose_{IV} / AUC_{INF,\,IV} * Dose_{PO}$

Pharmacokinetic Abbreviations

AUC_{INF}	Total AUC up to the last measurable concentration plus the AUC extrapolated from the last measurable concentration (C_{last} at t_{last}) to infinity: $AUC_{0\text{-}tlast} + C_{last}$/Lambda z
AUC_{last}	Area under the curve from the time of dosing to the time of the last measurable concentration
BLQ	Below the limit of quantitation
C_0	Instantaneous blood concentration following IV administration
Cl	Observed clearance
C_{max}	The maximum observed concentration
CV%	Percent coefficient of variation
F	Absolute bioavailability
N	Number
NA	Not Applicable
SD	Standard Deviation
T_{max}	The time at which C_{max} occurred

T_{last} Time of the last point with quantifiable concentration

$T_{1/2}$ Half-life

Vz Apparent volume of distribution as a function of bioavailability

Final Disposition

Study animals were not scheduled for sacrifice and were returned to the stock colony following the completion of the study.

Evaluation of the toxicity of gp120-targeting HIV entry inhibitor NBD-14189 in SCID-hu Thy/Liv mice

Table S1. Mann-Whitney U Test on treatment comparison data of NBD-14189 at different doses for toxicity evaluation in SCID-hu Thy/Liv mice.

**TREATMENT COMPARISON**	*P* VALUES								
**Group D (untreated)**	$\%CD4^+ CD8^+$	$\%CD4^+$	$\%CD8^+$	ratio 4/8	W6/32	%Live thymocytes	Total live cell yield	Live thymocyte yield	Body weight change
Group A: NBD-14189 300 mg/kg/day	0.729	0.643	0.203	0.118	**0.008**	0.643	>0.999	0.908	0.401
Group B: NBD-14189 100 mg/kg/day	0.488	0.224	0.165	0.133	0.247	0.643	0.247	0.247	0.674
Group C: NBD-14189 3 mg/kg/day	0.105	0.488	0.563	0.488	0.165	0.729	0.729	0.643	0.093

Evaluation of the antiviral activity of gp120-targeting HIV entry inhibitor NBD-14189 against HIV-1 NL4-3 in SCID-hu Thy/Liv mice

Table S2. Mann-Whitney U Test on treatment comparison data of NBD-14189 at different doses for antiviral efficacy evaluation in SCID-hu Thy/Liv mice.

**TREATMENT COMPARISON**	*P* VALUES												
**Group E (untreated, NL4-3-infected) versus:**	p24	HIV-1 RNA	%Gag-p24$^+$ thymocytes	%CD4$^+$ CD8$^+$	%CD4$^+$	%CD8$^+$	ratio 4/8	W6/32	%Live thymocytes	Total cell yield	Live thymocyte yield LSR II	Live thymocyte yield MUSE	Body weight change
Group A: NBD-14189 300 mg/kg/day, NL4-3	**0.004**	**0.004**	**0.016**	0.298	0.936	0.093	0.423	**0.025**	0.262	0.109	0.150	0.200	0.749
Group B: NBD-14189 100 mg/kg/day, NL4-3	**0.006**	**0.006**	0.100	0.201	0.855	0.273	0.855	0.144	**0.018**	0.465	0.144	0.465	**0.019**
Group C: NBD-14189 30 mg/kg/day, NL4-3	0.273	**0.006**	0.715	0.201	0.584	0.465	0.361	0.715	**0.018**	**0.018**	**0.018**	0.083	**0.029**
Group D: TDF/FTC 60/40 mg/kg/day, NL4-3	**0.004**	**0.004**	**0.016**	**0.010**	0.749	**0.007**	**0.037**	**0.007**	**0.004**	**0.007**	**0.004**	**0.004**	0.262
Group F: untreated, mock-infected	**0.006**	**0.006**	**0.029**	**0.006**	0.201	**0.006**	0.361	**0.006**	**0.014**	0.100	**0.018**	**0.018**	0.150
